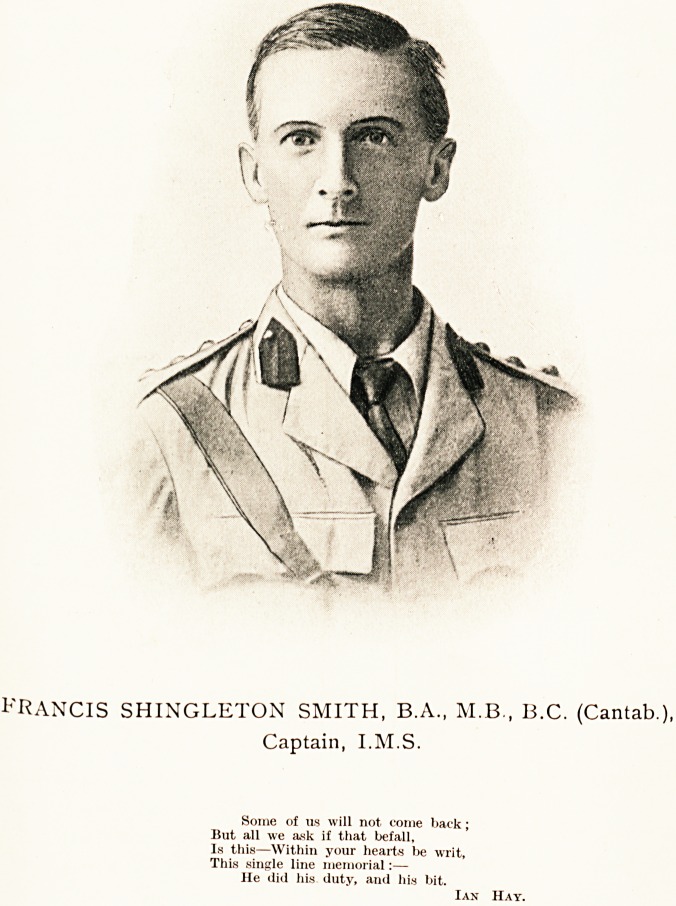# Francis Shingleton Smith

**Published:** 1915-12

**Authors:** 


### FRANCIS SHINGLETON SMITH, B.A., M.B., B.C. Cantab., Captain, I.M.S.

We deeply regret to record that Francis Shingleton Smith was reported killed in action on November 22nd, at the age of 36. Educated at Clifton College, he proceeded to King's College, Cambridge, in 1898, and after taking the B.A., continued his medical training in the Bristol University College and Bristol Royal Infirmary. Shortly after obtaining the degrees of M.B., B.C. Cambridge, he entered the Indian Medical Service in July, 1907.

He did duty in many districts, went with the relief column to Chitral, after which he was laid up at Calcutta with typhoid fever, which he had once before in Clifton. He made a good recovery, was appointed to Captain's rank in July, 1910, and was then transferred to the central provinces. In 1913 he was attached to the 120th Rajputana Infantry. He was home on leave in 1914, but when the war broke out was called back immediately, and received notice to be ready for foreign service.

In due course the regiment found itself at sea, and then bound for the Persian Gulf, where his many letters from Basra and Kurna described the ways of the Arabs of that region, for whom he had nothing good to say. At the battle of Sheiba some months later he was much interested in the excellence and efficiency of the artillery fire of our Indian troops, who cleared out trench after trench of the Turks with great precision.

After this he was sent into hospital at Basra for otorrhœa, apparently contracted by swimming in the Tigris, and at one time by diving for rifles dropped by the troops in swimming some of the creeks of the River Tigris. Then he had an attack of sand-fly fever, which lasted some fourteen days and caused much exhaustion, so much so that he was sent back to Bombay on sick leave. On the way thither he had charge of some of the Turkish prisoners bound for Rangoon: hitherto for him the Arab and Turk spoke no known language, but the doctors among these prisoners were able to speak French, and so he found that the Turkish doctors were good fellows whose companionship was quite agreeable.

On arrival at Bombay he was sent off to recoup at Kasauli, so exchanging the heat of Basra (120° shade) for the cool view of the Himalayan snows. On his return to the Gulf later in September the climate had become tolerable, pleasantly warm by day and a little cold at night. His companions on the transport were 1,200 Punjaubi recruits, who had never seen the sea, and who were overjoyed with the gambols of the porpoises in the Indian Ocean.

We cannot follow him up the river towards Baghdad, but the telegram from the Indian Office, received November 28th, gave his friends the unexpected information that he had been officially reported killed in action. It was reported later that his death took place at the battle of Ctesiphon, November 22nd—24th, and that it was instantaneous. One of his Indian colleagues writes: "You know that if it was to be, it was the

FRANCIS SHINGLETON SMITH, B.A., M.B., B.C. (Cantab.),
Captain, I.M.S.

Some of us will not come back;
But all we ask if that befall,
Is this—Within your hearts be writ,
This single line memorial:—
He did his duty, and his bit.

IAN HAY.

end he would have chosen, to die along with the regiment he had served so well and so long out there. Only the other day I heard how absolutely fearless and indefatigable Frank had shown himself, for I can just see him always busy, always ready to help and always cheery."

His many warm friends will feel that the writer evidently knew Frank Shingleton Smith, and has admirably summed up his main characteristics, for he was altogether unselfish, strong and lovable.

Shingleton Smith had a good knowledge of the numerous varieties of the mosquito as found in India, and regretted that he could not fully investigate the varieties found in the Persian Gulf, but he believed that they all differed somewhat from those found in Egypt and in India, being intermediate between the two.

He was the youngest son of Dr. Shingleton Smith, who till recently edited this *Journal*, and we offer our sincerest sympathies to Dr. and Mrs. Shingleton Smith and other members of his family in their loss.

We hope in our next issue to publish a posthumous paper by Capt. Shingleton Smith dealing with the "Forensic Investigation of Blood-stains."

## Figures and Tables

**Figure f1:**